# What do verbal fluency tasks measure? Predictors of verbal fluency performance in older adults

**DOI:** 10.3389/fpsyg.2014.00772

**Published:** 2014-07-22

**Authors:** Zeshu Shao, Esther Janse, Karina Visser, Antje S. Meyer

**Affiliations:** ^1^The Psychology of Language Department, Max Planck Institute for PsycholinguisticsNijmegen, Netherlands; ^2^Donders Institute for Brain, Cognition and Behaviour, Radboud UniversityNijmegen, Netherlands; ^3^Centre for Language Studies, Radboud UniversityNijmegen, Netherlands

**Keywords:** verbal fluency, lexical access, vocabulary knowledge, updating ability, inhibition

## Abstract

This study examined the contributions of verbal ability and executive control to verbal fluency performance in older adults (*n* = 82). Verbal fluency was assessed in letter and category fluency tasks, and performance on these tasks was related to indicators of vocabulary size, lexical access speed, updating, and inhibition ability. In regression analyses the number of words produced in both fluency tasks was predicted by updating ability, and the speed of the first response was predicted by vocabulary size and, for category fluency only, lexical access speed. These results highlight the hybrid character of both fluency tasks, which may limit their usefulness for research and clinical purposes.

## Introduction

The verbal fluency test is a short test of verbal functioning (e.g., Lezak et al., 2012). It typically consists of two tasks: category fluency (sometimes called semantic fluency; Benton, 1968) and letter fluency (sometimes called phonemic fluency; Newcombe, 1969). In the standard versions of the tasks, participants are given 1 min to produce as many unique words as possible within a semantic category (category fluency) or starting with a given letter (letter fluency). The participant's score in each task is the number of unique correct words.

Verbal fluency tasks are often included in neuropsychological assessment, in clinical practice, and in research. For instance, they have been used to support diagnoses of attention-deficit/hyperactivity disorder (ADHD; Andreou and Trott, 2013) and cognitive impairment in persons with neurodegenerative diseases, such as Alzheimer's disease (Zhao et al., 2013) or Parkinson's disease (Pettit et al., 2013). Verbal fluency tasks have also been used in research on non-clinical groups to measure verbal ability including lexical knowledge and lexical retrieval ability (e.g., Cohen et al., 1999; Weckerly et al., 2001; Federmeier et al., 2002, 2010) and as a test of executive control ability (e.g., Henry and Crawford, 2004; Fitzpatrick et al., 2013).

The wide-spread use of the verbal fluency tasks probably stems in part from their face validity as tests of both verbal ability and executive control: Participants need to retrieve words of their language, which undoubtedly requires them to access their mental lexicon, and they need to focus on the task, select words meeting certain constraints and avoid repetition, which certainly involves executive control processes (e.g., Fisk and Sharp, 2004). Thus, serious deficits in either verbal ability or executive control should manifest themselves in poor performance in the fluency tasks. Therefore, the fluency tasks can be used as an efficient screening instrument of general verbal functioning.

The validity of the fluency tasks as a tool to assess verbal ability (VA), specifically lexical access ability, has been confirmed in numerous studies comparing groups of participants that would be expected to differ in this ability. Lexical access ability is the ability to retrieve the grammatical representations and sound forms of words from the mental lexicon (e.g., Levelt et al., 1999). For instance, Sauzéon et al. (2011; see also Salthouse, 1991, 1996) found that participants with smaller vocabularies produced fewer words than did participants with larger vocabularies. Similarly, children with Specific Language Impairment or dyslexia, who often have word finding difficulties (e.g., Snowling et al., 1988; Seiger-Gardner and Brooks, 2008; Bragrad et al., 2012), have been shown to have deficits in verbal fluency performance compared to typically developing children (e.g., Cohen et al., 1999; Weckerly et al., 2001).

The validity of the fluency tasks as a tool to assess executive control ability (EA) is also well documented. Executive control is a set of functions that regulate one's thoughts and direct behavior toward a general goal. According to Miyake et al. (2000; see also Miyake and Friedman, 2012), three key components of executive control can be distinguished, namely updating (constant monitoring and tracking of working memory representations), shifting (flexibly switching between tasks or mental sets), and inhibition of dominant responses. Evidence for the involvement of aspects of EA in verbal fluency performance comes, for instance, from studies showing that children with ADHD had lower scores in verbal fluency tasks than typically developing controls (e.g., Mahone et al., 2001; Takács et al., 2013), and from studies demonstrating that damage to frontal brain areas is associated with poor performance in the fluency tasks (e.g., Baldo and Shimamura, 1998; Schwartz and Baldo, 2001).

However, it is not clear which components of executive control most strongly impact performance in the fluency tasks. To perform the task, participants must keep the instructions and the earlier responses in working memory and they must suppress irrelevant responses and repetition. Moreover, participants often produce sets of related words in succession (e.g., first name some pets, then switch to farm animals, then to birds), which involves the ability to create clusters based on a systematic memory search and the ability to alter the search criteria an switch from one category to the next. Henry and Crawford (2004) suggested that verbal fluency performance reflected working memory (see also Rosen and Engle, 1997; Rende et al., 2002), inhibition (see and Hirshorn and Thompson-Schill, 2006) and effortful self-initiation. Other authors have stressed the importance of switching ability (Abwender et al., 2001), which may, however, in its turn depend on response suppression processes (Hirshorn and Thompson-Schill, 2006). Verbal fluency has also been shown to be closely linked to fluid intelligence (e.g., Roca et al., 2012).

In sum, verbal fluency tasks are widely used because they afford rapid and reliable assessment of both VA and EA (e.g., Ettenhofer et al., 2006). However, the hybrid nature of the fluency tasks implies that verbal fluency scores are not a pure measure of either VA or EA. For the use of the fluency tasks in research contexts (for instance to match participants in two groups for VA or EA) and in clinical assessment, it would be useful to know how strongly the performance in each of the fluency tasks is affected by VA and EA.

Though the letter and category fluency task are obviously very similar, they do differ in subtle but important ways in task demands. The category fluency task resembles everyday production tasks, such as making a shopping list, so that participants can exploit existing links between related concepts (e.g., between the category label and the category members and among associated category members) to retrieve responses. By contrast, in the letter fluency task, words must be retrieved from a phonemic category, which is rarely done in everyday speech production, so that participants must suppress the activation of semantically or associatively related words and must resort to novel retrieval strategies (e.g., Luo et al., 2010; Katzev et al., 2013).

In line with these considerations, clinical studies have sometimes found marked discrepancies in patients' performance on the two tasks (e.g., Henry and Crawford, 2004). For instance, patients with Alzheimer's disease and psychosis have been observed to be relatively more impaired in the category than the letter fluency tasks, consistent with impaired access to semantic information (e.g., Laws et al., 2010; Magaud et al., 2010; Meijer et al., 2011). A similar profile has been seen in patients with focal lesions in areas implicated in access to semantic knowledge (e.g., Jones et al., 2006). Meanwhile, patients with amyotrophic lateral sclerosis have been observed to be more impaired in letter fluency task compared to controls and have reduced activation in the dorsolateral prefrontal cortex as an index of executive dysfunction (Quinn et al., 2012).

Consistent with these clinical observations, neuroimaging studies with healthy participants have demonstrated the involvement of overlapping, but not identical brain circuits, in the two tasks. Category fluency was found to be associated with activation more anterior-ventrally in left inferior frontal gyrus; whereas letter fluency is represented more posterior-dorsally in left inferior frontal gyrus (Costafreda et al., 2006; Robinson et al., 2012; Katzev et al., 2013), and in pre-supplementary motor area and left caudate (Grogan et al., 2009). In sum, the clinical and neuroimaging evidence suggests that verbal ability may be more strongly reflected in category than in letter fluency scores, and that, conversely, executive ability may be more strongly reflected in letter fluency scores.

In the study that is most directly related to the current work, Luo et al. (2010) showed that additional information about the contributions of verbal and executive control abilities to verbal fluency performance could be gleaned by recording not only the number of correct responses, but also the timing of the responses, specifically the time to produce the first response (1st RT hereafter) and the mean subsequent response time. The latter measure is the average time between the onset of the first response and the onset of each following response. It corresponds to the time when half the responses have been produced. A short mean subsequent RT indicates a fast declining rate of retrieval because a large proportion of the responses are produced early during the trial. Evidently, all measures, i.e., 1st RT, mean subsequent RT, and the resulting total number of words produced, depend on lexical knowledge and lexical access speed as well as executive control ability. However, Luo and colleagues proposed that the impact of EA should increase across the trial because participants need to remember earlier responses, suppress interference from these responses, and shift between subgroups of words (e.g., from nouns to verbs starting with a given letter). Therefore mean subsequent RT should be more sensitive to EA than the 1st RT.

Luo et al. (2010) compared the fluency task performance of English monolingual speakers and of two groups of bilingual speakers differing in English vocabulary size. They computed the total number of responses, 1st RT and subsequent mean RTs and carried out time course analyses of the retrieval process. They found that the three groups did not differ in performance in the category fluency task. However, in the letter fluency task, the high-vocabulary bilingual speakers produced more responses than the remaining two groups, and both groups of bilingual speakers had longer subsequent RT than the monolingual speakers had. Luo et al. concluded that these results indicated that the bilingual speakers had better EA than the monolingual speakers.

The study by Luo et al. (2010) was carried out with young bilingual speakers. In the present study we examined the verbal fluency performance profiles of older speakers with Dutch as their dominant language. This study aims to determine whether or not the two fluency tasks differed in their sensitivity to verbal and executive control ability within a group of older persons. Testing older adults has two advantages. First, groups of older adults are generally more heterogeneous than younger groups in terms of EA (cf. Ardila, 2007), which increases the chance of observing relationships between verbal fluency performance and EA predictors. Second, testing older adults is important because verbal fluency tasks are often used in clinical and research contexts to assess VA or EA of patients after stroke, patients with Alzheimer's disease or Parkinson's disease. These patients are often in their sixties and older, and information about the performance of healthy persons in the same age range and about the skills tapped in the fluency tasks should be useful to both clinicians and researchers.

In addition, age has been shown to impact verbal fluency performance. For instance, Bolla et al. (1990) found that age, verbal IQ, and gender were predictors of performance in verbal fluency tasks. Several studies showed an age-related decrease in the total number of words produced in the category fluency task (e.g., Crossley et al., 1997; Troyer et al., 1997; Troyer, 2000). Inconsistent effects have been reported, however, for aging effects on letter fluency. Some researchers showed an age-related decrease in the total number of words produced (e.g., Brickman et al., 2005; Rodriguez-Aranda and Martinussen, 2006), whereas other researchers reported that performance was stable across the tested age range (e.g., Crossley et al., 1997; Troyer et al., 1997). However, most of previous studies did not control VA and EA while measuring the age effect. In the present study, we investigated whether, within a group of older adults, age explained any unique variance in the category and letter fluency performance beyond any age differences in VA and EA measures.

Although Luo et al. (2010) made a strong case for the involvement of VA and EA in the fluency tasks, they did not correlate these abilities with the scores obtained in the fluency tasks. By contrast, in the present study participants completed separate tests of verbal and executive control skills in addition to the fluency tasks. Analyses of the correlations between the test scores allowed us to estimate how much linguistic and executive control skills contributed to performance in the fluency tasks.

Verbal skills were assessed in two ways: through a multiple-choice test of vocabulary size and a speeded picture naming task. However, picture naming is a hybrid task as well, depending on lexical access speed and executive control. Using ex-Gaussian analyses (see below for details), Shao et al. (2012) decomposed the mean naming RT into the normal (Gaussian) part of the underlying RT distribution (parameter μ) and the tail end of the RT distribution (parameter τ). Their results showed that updating (assessed through an operation span task) and inhibition abilities (assessed through a stop-signal task) correlated with the parameter τ but not μ. This suggests that μ of the RT distribution reflects general lexical access speed and τ reflects the influence of executive control: Poorer executive control leads to relatively more very slow responses. In the present study, we also carried out ex-Gaussian analyses of the picture naming RT and used the parameter μ to estimate lexical access speed, and τ to estimate executive control abilities.

In addition, updating and inhibition were assessed in separate tests. We focused on these two components of executive control because, as mentioned above, they have been shown to be involved in word production using a picture naming task (Shao et al., 2012), and because their involvement in the fluency task has been suggested repeatedly in the literature (e.g., Henry and Crawford, 2004; Laws et al., 2010), but never been assessed in an individual differences study. Updating may be involved in verbal fluency tasks because participants need to retrieve words within a given semantic category or starting with a particular phoneme and keep track of words that have already been produced to avoid repetition. In the present study, updating was measured using the operation span task (Turner and Engle, 1989), which requires participants to solve mathematical problems while memorizing lists of words. We used the operation span task because it measures the ability to store and regularly keep track of relevant information while conducting another cognitive task. Inhibition may be involved in the fluency tasks to suppress activation of repeated and/or irrelevant non-matching responses (e.g., Hirshorn and Thompson-Schill, 2006). We used a stop-signal task, which is widely used as a non-linguistic task to measure inhibition ability (e.g., Miyake et al., 2000). It measures how fast a person can withhold a planned response. As mentioned above, in Shao et al. (2012), we found a positive correlation between stop-signal RT and picture naming RT, suggesting that general inhibition ability is involved in picture naming. Therefore, if inhibition is involved in verbal fluency as in picture naming, correlations between stop-signal RT and indicators of verbal fluency should be seen.

If VA and EA variables affect verbal fluency performance in similar ways in different speaker groups of participants (in younger and older people; in bilingual and monolingual speakers), the patterns of results seen in the present study should be consistent with those observed by Luo et al. (2010). Therefore, we expected, first, vocabulary knowledge and lexical access speed to predict the number of responses in the fluency tasks and the speed of the first response. Second, we expected the indicators of executive control to predict the number of correct responses and the mean RT of the following responses. Finally, we expected variables related to lexical access ability to be relatively more important for category fluency performance than for letter fluency performance and, conversely, variables related to executive control ability to be relatively more important for letter fluency than for category fluency performance.

## Method

### Participants

The study was carried out with 82 participants (30 men^1^) whose mean age was 71.77 years. Seventeen participants were aged between 60 and 69 years, 36 between 70 and 79 years, and 8 between 80 and 89 years. Participants were paid €8 per hour for their participation. All participants were Dutch native speakers with normal or corrected-to-normal vision and only two of them were bilingual speakers. Their visual acuity was tested using a Landolt chart. All participants scored above 1.5 point.

All participants were presented with the tasks in the following order: picture naming, verbal fluency, operation span, and stop-signal task. Their vocabulary was tested in a different session, in the context of another study (Janse and Adank, 2012; Scharenborg and Janse, 2013; Janse and Jesse, 2014). All participants provided informed consent before the experiment and their data were analyzed anonymously. The educational background of participants was expressed on a scale from 1 (primary school education) to 6 (university education). The average educational level was high school education (*Mean* = 4; *SD* = 1.68)^2^.

### Verbal fluency tasks

#### Materials and procedure

On each trial, participants were asked to overtly generate as many Dutch words as possible within 60 s. First, category fluency was assessed in two trials: participants were asked to produce words in the animal category and then in the food and drink category. Letter fluency was assessed in the next two trials: participants were first asked to produce words that start with the letter M and then with the letter S. Each new trial was triggered when participants pressed the space bar. Then the category or letter was presented in the center of the screen with a horizontal white bar on the bottom of the screen. The white bar represented an hourglass as it shrunk during 1 min until it disappeared. Participants were asked to avoid producing names of people or places and repetitions of words.

#### Data analysis

Repetitions of the same word and proper names of people and places were excluded from the data analysis. The total number of words was calculated for each trial and for each participant and was then averaged separately for the category and letter fluency tasks (mean category and letter fluency score hereafter). The timing of the responses was analyzed following Luo et al. (2010). Specifically, we computed the onset of the first word retrieved in a trial (1st RT) and the mean of the time interval between the onset of the first retrieved word and each subsequently retrieved word (hereafter mean subsequent RT). As shown by Luo et al. (2010; see also Rohrer et al., 1995), the number of produced words declines as a function of time, such that generally more words are produced at the beginning than toward the end of the 60 s period (i.e., an exponentially declining pattern). According to Rohrer et al. (1995), mean subsequent RT is a better indicator of retrieval decline during free recall than the average of response RT (the average time lag between response onset to task onset) because the decline rate is exponential (cf., Figure 2 in Rohrer et al., 1995). Mean subsequent RT indicates the time point when 50% of the responses have been given. A short subsequent RT indicates a fast decline rate in retrieval speed.

### Picture naming task

#### Materials

One hundred and sixty-two line-drawings of objects were used for the naming task. All pictures were adapted from an English naming battery (Druks and Masterson, 2000) and had been used in an earlier study with Dutch speakers (Shao et al., 2013a). The frequency of the object names covered a broad range (log frequency 0–3.58), with an average log frequency of 1.43 (*SD* = 0.61) according to the SUBTLEX-NL database (Keuleers et al., 2010). The dominant names of the pictures were the names used by the majority of the participants in the present study. All pictures were scaled to fit into frames of 4 by 4 cm on the participant's screen (2.29° of visual angle).

#### Procedure

The procedure followed Shao et al. (2012). On each trial, a fixation cross (+) was presented for 800 ms in the center of the screen. Then a picture was shown for 600 ms, followed immediately by a red flashing exclamation mark that was presented for maximally 1400 ms. Participants were encouraged to name the pictures before the onset of the red exclamation mark but had the full 2 s interval to respond. The intertrial interval was 1500 ms. A trial ended when the voice key was triggered by the participant's verbal response or automatically 2 s after picture onset if the participant did not respond. Four pseudo-randomized testing lists were used, which were counterbalanced across participants.

#### Apparatus

The study was carried out using a HP 8540P laptop with the Presentation® software package (Version 14.3, www.neurobs.com). Naming RTs were recorded online using a voice key and were manually checked later using the speech analysis program Praat (Boersma, 2001).

#### Data analysis

Responses were coded as errors when participants used names different from the dominant names of the pictures, or when the responses included a repair or started with a stutter or a filler word (e.g., “uh”). Errors were excluded from the data analysis. As reaction times (RTs) are distributed asymmetrically, we decomposed the mean RTs into two components following an ex-Gaussian function: The leading edge (μ) and the tail (τ) of the underlying distribution. The parameter μ reflects the mean of the Gaussian portion and τ reflects the mean and standard deviation of the exponential portion. We used QMPE software (Brown and Heathcote, 2003) to derive the ex-Gaussian parameters for each participant.

### Operation span task

#### Materials

The operation span task was adapted from Shao et al. (2012; Turner and Engle, 1989). Sixty mathematical operations and words (translated into Dutch) were used as in Shao et al. (2012).

#### Procedure

On each trial, a fixation cross was presented for 800 ms followed by a blank interval of 100 ms. Then a combination of a mathematical operation and word was presented in the center of the computer screen in Arial 26 point font size [(3 ^*^ 4) − 3 = 9? River “river”]. Participants were asked to read the operation and word aloud, remember the word, and then indicate whether or not the operation was correct by pressing a response key (“/” key for correct operations and “Z” key for wrong operations). A recall cue (“Schrijf op” [write down]) was presented after a run of trials, which randomly varied from 2 to 6 trials. Participants were required to write down the remembered words in the sequence they were presented. The task was self-paced.

#### Data analysis

The operation-span score was calculated as the sum of words that were recalled in the right order on trials with correct responses to the mathematical operation. A participant's score could range from 0 to 60.

### Stop-signal task

#### Materials, design, and procedure

To assess the ability to inhibit responses, we used the stop-signal task Stop-it (Verbruggen et al., 2008). The visual stimuli were a square (1.5 by 1.5 cm) and a circle (1.5 cm in diameter). The auditory stimulus was a 750 Hz tone lasting for 75 ms. The output volume of the computer speaker was set to a fixed loud and clear presentation level. On go trials, a fixation cross (+) was presented in the center of the screen for 250 ms and followed by the visual stimuli (i.e., square or circle) for a maximum of 1250 ms. All visual stimuli were presented equally frequently in a random order. The participants were instructed to press the “/” key when they saw a circle and the “Z” key when they saw a square as quickly as possible. The trial was terminated by a key press. On no-go trials, the tone was played as a stop signal shortly after the onset of the visual stimulus, indicating that the participant should withhold the response. The stop-signal delay (SSD; i.e., the time interval between offset of fixation cross and onset of stop signal) was initially set to 250 ms. If the participant successfully inhibited the response on a given stop trial, the SSD in the next stop trial was increased by 50 ms, otherwise it was decreased by 50 ms. This increase or decrease of SSD was progressive and in principle the SSD could vary from 0 ms (when responses on all previous stop trials were correctly inhibited) to 1000 ms (when responses on all previous stop trials failed to be inhibited).

There was a practice block of 32 trials, followed by three test blocks of 64 trials each. Each block contained 75% go trials and 25% no-go trials, presented in a random order. Following Verbruggen et al. (2008), we estimated each participant's stop-signal reaction time (SSRT) by subtracting the mean SSD across all trials from the mean RT on go trials. Short SSRTs indicate good inhibitory control in that participants can stop their responses relatively late during response preparation.

### Vocabulary test

Vocabulary knowledge was assessed by a receptive multiple choice test (Andringa et al., 2012). This test consists of 60 multiple-choice questions, where participants have to indicate which of four descriptions best matched the “difficult word” (a fifth option always being “I really don't know”). For instance, for the Dutch word *mentaliteit* (mentality), the (translated) carrier phrase is “What a strange mentality!,” and participants choose from the following options (translated): (1) table, (2) person, (3) way of thinking, (4) atmosphere, (5) I really don't know. The proportion of correct items (out of 60) was used to index participants' vocabulary knowledge. This vocabulary test has not been standardized, but scores have been shown to predict general listening performance (Janse and Jesse, 2014) and adaptation to a foreign-sounding artificial accent (Janse and Adank, 2012). Furthermore, as part of a general Linguistic Knowledge construct, the scores have been shown to be the most important predictor of text comprehension in both native and non-native listeners (Andringa et al., 2012).

## Results

The results obtained from eight participants were excluded from the following analyses because of poor performance on the operation span task (less than 85% correct response on the mathematic operations; a criterion suggested by Turner and Engle, 1989). Table 1 summarized the performance in all tasks in the current study and relevant descriptive information from previous studies (Luo et al., 2010 and Shao et al., 2012).

**Table 1:** **Mean score, the 1st reaction time (RT) and subsequent RT in the category and letter fluency task, mean score and error rate of operation span task, stop-signal RT (SSRT) and mean RT of go trials of stop-signal task, mean naming RT, ex-Gaussian parameters and error rate of picture naming task**.

**Task**	**Performance**					
	**The present study**		Luo et al., 2010		Shao et al., 2012	
	**Mean**	***SD***	**Mean**	***SD***	**Mean**	***SD***
**CATEGORY FLUENCY**						
Mean score	22	5.8	21	4.2	–	–
1st RT (ms)	4000	2430	1800	1400	–	–
Subsequent RT (ms)	26,240	3630	24,000	3300	–	–
**LETTER FLUENCY**						
Mean score	15	5.7	12	3.5	–	–
1st RT (ms)	2770	1110	1800	1000	–	–
Subsequent RT (ms)	26,570	3970	24,000	3600	–	–
**OPERATION SPAN**						
Mean score	29	8	–	–	43	9
Error rate (%)	7.3	4.3	–	–	–	–
**STOP-SIGNAL**						
SSRT (ms)	318	58	–	–	279	50
Go trial RT (ms)	673	113	–	–	–	–
**PICTURE NAMING**						
Mean RT (ms)	821	82	–	–	705	69
Ex-Gaussian μ	636	53	–	–	545	45
Ex-Gaussian σ	60	23	–	–		
Ex-Gaussian τ	182	55	–	–	160	35
Error rate (%)	16	15	–	–	11	–
**VOCABULARY TEST**						
Accuracy (%)	85.2	16.4	–	–	–	–

*SD, Standard Deviation*.

As shown by Table 1, verbal fluency scores and subsequent RTs in the current study were similar to those reported by Luo et al. (2010), whereas the 1st RTs were slightly longer. The RTs of the stop-signal task and the picture naming task were slower than those in the previous study testing students (Shao et al., 2012). Generally, older participants showed more variable performance than young participants used in previous studies (see *SD*s in Table 1). The average vocabulary score in the present study was somewhat higher than the score obtained by Andringa et al. (2012) for monolingual speakers (*Mean score* = 68%, *SD* = 6.3).

In order to examine the similarity between the category and letter fluency task performance, we correlated the three indicators (mean score, 1st RT and mean subsequent RT) across tasks. We found a significant correlation for the mean scores of the two tasks (*r* = 0.65, *p* < 0.001), but not for 1st RT of the two tasks (*r* = 0.10, *p* = 0.42), or the mean subsequent RT (*r* = 0.09, *p* = 0.46).

Then we investigated the inter-correlations among the predictor variables: participants' performance on the picture naming task (quantified by the ex-Gaussian parameters μ and τ of their naming RT distribution), vocabulary score, operation span, and stop-signal task performance, as well as their age (in years). The results are shown in Table 2. Age was related to most measures, except vocabulary size, which was only related to operation span. Consistent with findings by Shao et al. (2012), we found that naming RT was negatively related to operation span score and positively related to SSRT.

**Table 2:** **Relationships between operation span score (OSPAN), stop-signal response time (SSRT), age, and naming RT and ex-Gaussian parameters μ and τ for picture naming**.

	**Naming RT**	**Naming μ**	**Naming τ**	**OSPAN**	**SSRT**	**Age**
Vocabulary	0.09	0.08	0.02	0.21^*^	−0.06	−0.10
Naming RT		0.77^**^	0.76^**^	−0.22^*^	0.19	0.39^**^
Naming μ			0.18	−0.23^*^	0.21^*^	0.39^**^
Naming τ				−0.11	0.07	0.22^*^
OSPAN					−0.04	−0.27^**^
SSRT						0.20^*^

* p < 0.05;

** *p < 0.01*.

Next we correlated participants' performance on the verbal fluency tasks (indicated by total number of correct responses, 1st RT and mean subsequent RT) with the vocabulary score, lexical access speed (ex-Gaussian parameters μ), the indicators of executive control (ex-Gaussian parameter τ, OSPAN score, and SSRT), and the participant's age. The correlations are listed in Table 3 and will be commented upon in the Discussion.

**Table 3:** **Relationships between the indicators (mean score, 1st and subsequent response time) of category and letter fluency performance and the individual measures (OSPAN score, SSRT, and naming response time and ex-Gaussian parameters μ and τ, vocabulary performance) and age (in years)**.

**Task**		**OSPAN**	**SSRT**	**Naming RT**	**Naming μ**	**Naming τ**	**Vocabulary performance**	**Age**
Category	Mean score	0.46^**^	−0.14	−0.39^**^	−0.29^**^	−0.30^**^	0.20^*^	−0.41^**^
	1st RT	−0.21^*^	0.06	0.33^**^	0.32^**^	0.21^*^	−0.26^*^	0.34^*^
	Subsequent RT	−0.21^*^	0.14	0.21^*^	0.27^*^	0.07	0.12	−0.01
Letter	Mean score	0.45^**^	−0.13	−0.34^**^	−0.27^*^	−0.27^*^	0.06	−0.32^*^
	1st RT	−0.11	0.02	0.12	0.17	0.01	−0.27^*^	0.07
	Subsequent RT	−0.04	−0.03	0.04	−0.11	0.15	−0.02	−0.07

** p <0.01;

* *p <0.05*.

Finally, we carried out multiple regressions to examine the independent contribution of each predictor to the prediction of verbal fluency performance. All predictors were entered into the model simultaneously. The dependent variables to be accounted for were the mean fluency scores, 1st RT, and average subsequent RT of the category and letter fluency task. The predictor variables were the operation span score, vocabulary score, picture naming parameters μ and τ, and age. SSRT was not included because it was not related to any performance indicators of the verbal fluency tasks. Tables 4**–6** show the multiple regression results. Updating ability significantly predicted the mean score of both verbal fluency tasks and the mean subsequent RT of the category fluency task (see Table 4). The vocabulary score significantly predicted the first RT of both verbal fluency tasks (see Table 5). Average lexical access speed (parameter μ) contributed significantly to the 1st and average subsequent RTs of the category fluency tasks (Tables 5, 6). Parameter τ contributed little to predicting any performance indicator of the fluency tasks.

**Table 4:** **Results of the multiple regression analysis with mean scores of category and letter fluency tasks as criterion variables and vocabulary knowledge, ex-Gaussian parameter μ and τ of naming response time, operations span (OSPAN), and age as predictor variables**.

	**Category**				**Letter**			
	***B***	***SE* of *B***	***R^2^***	***F***	***B***	***SE* of *B***	***R^2^***	***F***
Vocabulary	4.41	3.58	0.36	7.53^**^	−0.50	3.70	0.28	5.32^**^
Naming μ	−0.01	0.01			−0.01	0.01		
Naming τ	−0.02	0.01			−0.02	0.01		
OSPAN	0.50^**^	0.16			0.27^**^	0.08		
Age	−0.21	0.10			−0.13	0.11		

*Unstandardized B, Standard Error of B, R^2^ and F-Value are listed*.

** p < 0.01;

^*^p < 0.05. Mean of Cook's distance = 0.02.

**Table 5:** **Results of the multiple regression analysis with 1st response time (RT) of category and letter fluency tasks as criterion variables and vocabulary knowledge, ex-Gaussian parameter μ and τ of naming RT, operations span (OSPAN), and age as predictor variables**.

	**Category**				**Letter**			
	***B***	***SE* of *B***	***R^2^***	***F***	***B***	***SE* of *B***	***R^2^***	***F***
Vocabulary	−3.79^*^	1.63	0.24	4.25^**^	−1.93^*^	0.81	0.11	1.66
Naming μ	0.01^*^	0.01			0.00	0.00		
Naming τ	0.01	0.01			−0.00	0.00		
OSPAN	−0.01	0.04			−0.00	0.02		
Age	0.07	0.05			−0.01	0.02		

*Unstandardized B, Standard Error of B, R^2^ and F-Value are listed*.

** p < 0.01;

* *p < 0.05. Mean of Cook's distance = 0.02*.

**Table 6:** **Results of the multiple regression analysis with subsequent response time (RT) of category and letter fluency tasks as criterion variables and vocabulary knowledge, ex-Gaussian parameter μ and τ of naming RT, operations span (OSPAN), and age as predictor variables**.

	**Category**				**Letter**			
	***B***	***SE* of *B***	***R^2^***	***F***	***B***	***SE* of *B***	***R^2^***	***F***
Vocabulary	0.65	2.56	0.15	2.49^*^	−0.65	2.97	0.05	0.68
Naming μ	0.02^**^	0.01			−0.01	0.01		
Naming τ	0.00	0.01			0.01	0.01		
OSPAN	0.13^*^	0.06			0.01	0.07		
Age	−0.04	0.07			−0.05	0.09		

*Unstandardized B, Standard Error of B, R^2^ and F-Value are listed*.

** p < 0.01;

* *p <0.05. Mean of Cook's distance = 0.01*.

## Discussion

Verbal fluency tasks are widely used to assess verbal functioning in clinical and research settings. This is because the tasks have compelling face validity: A person with a serious deficit in lexical access, executive control abilities or both will perform poorly in the tasks. While fluency scores are useful indicators of general verbal functioning, it is for many purposes important to understand how strongly performance in the tasks is affected by each of the abilities involved.

Luo et al. (2010) assessed this issue by comparing the fluency task performance of monolingual speakers and two groups of bilingual speakers differing in their second language vocabulary size. They used category and letter fluency tasks and introduced different performance indicators: the number of correct responses, the RT to retrieve the first response (1st RT) and the mean subsequent RT, which is the time when half of the responses have been given. As described above, they found that the three groups of participants did not differ in performance in the category fluency task. However, in the letter fluency task, the high-vocabulary bilingual speakers had a higher mean score than the monolingual group and both groups of bilingual speakers had longer subsequent RTs than the monolingual speakers had. These results support the view that bilingual speakers have better executive control ability than monolingual speakers (cf. e.g., Singh and Mishra, 2013, for more details on the locus of this bilingual cognitive control advantage), and that executive control ability is primarily expressed in performance in the letter fluency task.

Whereas Luo and colleagues compared the performance of different groups of young participants, we tested one group of older participants and assessed how well their performance in the fluency tasks, measured in the same way as in the study by Luo et al., correlated with independent indicators of verbal and executive control ability (VA and EA). VA was decomposed into vocabulary knowledge, assessed in a vocabulary test, and lexical access speed, estimated by the parameter μ of the participant's distribution of picture naming RT. Executive control was estimated using the parameter τ of the distribution of picture naming RT, the score of the OSPAN task (assessing updating), and the SSRT for the stop-signal task (assessing inhibition).

We expected that good vocabulary knowledge and fast lexical access should be related to good performance in the fluency tasks. Good performance in the executive control tasks should also be related to good performance in the fluency tasks. In addition, we expected that lexical access ability should have a stronger impact on performance in the category fluency than in the letter fluency task, and, conversely, that executive control ability should have a stronger impact on performance in the letter fluency, compared to the category fluency task. We will first consider the results of the analyses of correlations and then turn to the results of the regression analyses.

In the raw correlations of the predictors with the indicators of the fluency performance, we found some support for the predictions laid out above. The vocabulary score correlated positively with the mean scores in the category fluency task and negatively with the first RT in both fluency tasks. There was no significant correlation with the mean score in the letter fluency task, in line with the expectation that vocabulary size is more important in the category than in the letter fluency task. Furthermore, lexical access speed, estimated by the parameter μ of the picture naming task, correlated negatively with the mean scores in both tasks, and positively with the first and mean subsequent RTs in the category fluency task. There was no correlation with the first and mean subsequent RTs in the letter fluency task, further supporting the view that lexical access ability is a more influential determinant of performance in the category than in the letter fluency task.

Turning to executive control ability, τ of the distribution of picture naming RT was negatively correlated with the mean scores in both tasks, and positively correlated with the first RT in the category fluency task. It was, however, not correlated with the first RT in the letter fluency task or with the mean subsequent RT in either task. The OSPAN scores were positively related to the mean scores in both tasks, and they were negatively related with the first and mean subsequent RTs in the category fluency task, but not in the letter fluency task. Contrary to our expectations, SSRT (indicative of inhibition) was not correlated with any of the performance indicators of the fluency tasks. In considering this finding, it is important to note that inhibition is not a unitary function but consists of a set of closely related functions (e.g., Nigg, 2000; Castner et al., 2007; Spaulding, 2010). The stop-signal task measures how fast a person can stop a planned response. In the verbal fluency tasks, a different type of inhibition, selective inhibition may be more important. This is the ability to suppress the activation of highly potent competitors to a target response (see Shao et al., 2013b). Future research might investigate whether selective inhibition ability is reflected in verbal fluency performance.

Finally, in contrast to results obtained by Bolla et al. (1990; but see Crossley et al., 1997; Troyer et al., 1997), in our study age did not explain any unique variance in any indicator of verbal fluency performance. However, age was associated with the participants' scores on the OSPAN task, picture naming RT, and stop-signal RT, suggesting that age may have affected our results indirectly. It should be recalled that the present study only included older participants; it is an open empirical issue whether our findings would be replicated in samples with a broader age range.

Overall, the correlations suggest that vocabulary knowledge and lexical access speed are somewhat more important determinants of category than of letter fluency. However, there was no evidence that executive control had a stronger effect on performance in the letter than in the category fluency task. This is not entirely consistent with findings of Luo et al. (2010), which may be due to the differences in samples (younger vs. older persons; bilinguals vs. monolinguals) and research approach (group comparison vs. correlational approach). Future research is needed to investigate the reasons for the difference in the results of the two studies.

Since the indicators of VA and EA were not independent of each other and both types of indicators were affected by age, it is important to consider the results of the regression analyses in evaluating the independent effects of VA and EA on performance in the fluency tasks. In the next section, we discuss the results obtained for each dependent variable, i.e., the mean score, the first RT, and the mean subsequent RT.

As Table 4 shows, the only significant predictor of the mean scores of both fluency tasks was performance in the OSPAN task. OSPAN performance was expected to predict the performance in the fluency tasks, but, contrary to the prediction, it did not have a stronger effect on performance in the letter than in the category fluency task. Good updating ability apparently contributes to good performance in both fluency tasks. This is plausible as in both tasks participants have to keep activating new words within the given category or starting with the given letter, while keeping track of the words that have been produced to avoid repetitions. The finding that the mean score in both fluency tasks depended primarily on updating ability, rather than on vocabulary size or lexical access speed, is important as the mean score is sometimes used to measure language production ability (e.g., Cohen et al., 1999; Weckerly et al., 2001; Federmeier et al., 2010). However, our findings suggest that the fluency scores depend mainly on the ability to store and update relevant information in working memory, rather than verbal ability. It is, however, important to note that we assessed older adults speaking in their dominant language. The relative impact of updating and verbal abilities may be different in other groups of participants. An important challenge for research into individual differences in linguistic and cognitive skills is to determine which underlying abilities or traits have a pervasive influence on performance across different groups defined, for instance, by socio-economic or demographic factors, and which abilities and skills only affect performance of individuals in specific groups.

The only significant predictors of the first RT for both fluency tasks were the vocabulary score and, for category fluency only, the average speed of lexical access (parameter μ of the distribution of picture naming RT, see Table 5). Thus, there was evidence for the involvement of lexical access ability in the fluency tasks, but it was seen more clearly in the speed of the responses than in the overall scores. As predicted, the influence of lexical access ability was more consistent in the category than in the letter fluency task.

Finally, the mean subsequent RT in the category fluency task was predicted by lexical access speed and performance in the OSPAN task (see Table 6). The effect of both predictors was modest and roughly equal. By contrast, none of the predictors used here significantly predicted the mean subsequent RT in the letter fluency task. This difference in results seen for category and letter fluency may reflect the different retrieval mechanisms employed in the two tasks. Judging from the mean scores, the letter fluency task was harder than the category fluency task for the participants of the present study. As explained in Introduction, retrieval of a word (e.g., *cat*) will automatically activate semantically associated words (e.g., *dog*, *tiger*, *mouse*; Dell, 1986; Levelt et al., 1999). Thus, in performing the category fluency task participants can rely on existing links between concepts or words. The links between words beginning with the same letter may be weaker or less accessible so that novel search strategies may be required to carry out the task. This may have led to large variability in the time course of the retrieval of the responses across participants. In addition, the letter fluency may have engaged cognitive skills or abilities not tapped here (e.g., fluid intelligence, as proposed by Roca et al., 2012).

In sum, our results show, as expected that (a) performance on the fluency tasks was affected by both executive control ability and verbal ability, (b) that different performance indicators provided different information, in that they were relatively more, or less, affected by executive control and verbal ability, and (c) that category fluency performance was somewhat more strongly affected by verbal ability than letter fluency performance. In evaluating these findings, it is important to keep in mind that the participants of the present study were healthy older adults. The relative difficulty of the two fluency tasks may be different for younger participants (Meinzer et al., 2009) and for clinical samples. Our results do not put into question the suitability of the fluency tasks as a reliable diagnostic tool to determine exactly what the name suggests—a person's verbal fluency, their general verbal functioning. However, for many purposes, in particular in research contexts, it might be advisable to assess verbal and executive skills separately. Verbal knowledge can best be accessed in untimed vocabulary or grammar tasks, and speed of lexical access in naming and word recognition tasks. Executive control abilities can be assessed in verbal and non-verbal tasks tailored to measure specific components of executive control.

### Conflict of interest statement

The authors declare that the research was conducted in the absence of any commercial or financial relationships that could be construed as a potential conflict of interest.

## Footnotes

^1^Due to unequal numbers of male and female participants, the contribution of gender to the fluency scores was not investigated in the present study.

^2^Level of education did not correlate significantly with any of the predictors (*r*s < 0.13, *p*s > 0.10) and was not included in the analysis reported here.
